# What´s in the tank? Nematodes and other major components of the meiofauna of bromeliad phytotelms in lowland Panama

**DOI:** 10.1186/s12898-016-0069-9

**Published:** 2016-03-15

**Authors:** Gerhard Zotz, Walter Traunspurger

**Affiliations:** Functional Ecology Group, Institute of Biology and Environmental Sciences, University of Oldenburg, Box 2503, 26111 Oldenburg, Germany; Smithsonian Tropical Research Institute, Ancon, Apartado Postal 0843-03092, Balboa, Republica de Panama; Animal Ecology, University of Bielefeld, Konsequenz 45, 33615 Bielefeld, Germany

**Keywords:** Barro Colorado island, Bromeliaceae, Habitat size, Island biogeography, Nematodes, Phytotelms, Rotatoria, *Werauhia sanguinolenta*

## Abstract

**Background:**

Nematodes are a very diverse and extremely abundant group of animals, but their occurrence in the tropics is surprisingly little understood. We investigated the meiofauna of epiphytic tank bromeliads in the lowlands of Panama with particular emphasis on nematodes.

**Results:**

We encountered 89 morphospecies of nematodes in 54 bromeliad tanks, which were sampled in the wet and the dry season. Rotifers were by far the most abundant group in both the dry and the wet season (with up to 960 individual ml^−1^), followed by nematodes, annelids and harpacticoid copepods. Individual plants hosted up to 25 nematode species. These nematodes represented a diversity of feeding guilds, suction-feeders and deposit-feeders being most abundant. The relative abundances of feeding-types of nematodes differed considerably in the wet and dry season. Both species richness and abundance were strongly correlated with the size of the phytotelms and the season, while species diversity assessed with the Shannon-index was affected by neither of the two.

**Conclusion:**

This is the first study with a particular focus on the diversity of nematodes in tank bromeliads. We document a meiofauna of considerable abundance and diversity, which suggests important functional roles in ecological processes such as decomposition, which in turn warrants further study.

**Electronic supplementary material:**

The online version of this article (doi:10.1186/s12898-016-0069-9) contains supplementary material, which is available to authorized users.

## Background

Nematodes are a very diverse group with some 20,000 described species, although they are even more remarkable in terms of abundance than diversity: there are estimates that globally four out of every five multicellular organisms are nematodes [1]. Surprisingly, there are some indications that nematode communities in the tropics are less species-rich than those in temperate ecosystems, in contrast to the usual decrease in species numbers with latitude [2]. However, such a conclusion may be premature and simply represent a sampling artefact: (1) only 10 of 134 published ecosystem surveys analysed in the review of Boag and Yeates [3] were from tropical latitudes and this strong geographical bias has not changed since, and (2) the few species estimates for the tropics are undoubtedly underestimates because habitats above the mineral soil, e.g., canopy soils or phytotelms, are seldom, if ever sampled [2]. There are just a few scattered reports on nematodes in phytotelms such as tree holes or bamboo stumps [4–6], in organic material accumulated in epiphytic *Asplenium nidus* [7] or from pitcher plants [8]. In his review on animals in container habitat, Kitching [9] laconically states that nematodes have “received a very modest amount of attention”, which is still true a decade later.

Tank bromeliads are a major structural component in Neotropical forests [10, 11], and the biota inhabiting these natural microcosms have received considerable attention in recent years as attractive model system for numerous ecological questions [12–14]. Many studies focus on insects and other arthropods (e.g., [13, 15, 16]), but other groups are also studied, for example protists (e.g., [17, 18]), crustaceans [19], other invertebrate groups [20], or algae [21], while information on nematodes in bromeliad tanks is largely non-existent. The only exceptions are a study by Zullini et al. [22] from Costa Rica, which mentions two nematode species found in bromeliads, and a list of 12 species (from a single tank bromeliad) in a review on “extreme freshwater habitats” [23]. Nematodes also represent an important link between microbenthos and macrobenthos, e.g., they do increase the activity of bacteria and serve as food for many other benthic organisms, like crustaceans and chironomids [24, 25].

The surprising and blatant lack of information on the occurrence of nematodes and other meiofaunal organisms in these natural freshwater habitats—as the earth´s most abundant group of metazoans—motivated the current study, which addressed three major questions: (1) How diverse are the nematode assemblages in tank bromeliads, (2) does diversity differ between dry and wet seasons with fundamentally different moisture regimes in these habitats [26], (3) how much do abundances, species composition, and guild structure depend on the size of these container habitats?

Although focusing on nematodes, we also investigated—to a lesser degree—a number of other groups, which are generally underrepresented in the published studies with tank bromeliads, e.g., rotifers, annelids, or mites, but only registered abundances of each group, without distinguishing individual species.

## Methods

### Study site and sampling procedure

The study was carried out in the Barro Colorado Nature Monument (9°10′N, 79°51′W), Republic of Panama. The vegetation of this biological reserve is classified as a tropical moist forest [27]. Average annual rainfall is about 2600 mm, the average temperature is c. 27 °C. While there is very little annual variation in temperature, rainfall is highly seasonal—a pronounced dry season with rainless periods of up to several weeks lasts from late December to late April [28]. Detailed descriptions of vegetation, climate and ecology are reported elsewhere [29, 30].

A total of 54 individuals of four species of bromeliads were sampled: *Guzmania monostachia* (L.) Rusby ex Mez, *Tillandsia elongata* H.B.K. var. *subimbricata* (Bak.) L. B. Sm., *Tillandsia fasciculata* Sw. var. *fasciculata*, and *Werauhia sanguinolenta* (Linden ex Cogn. & Marchal) Grant, with a focus on the last species. The sampled plants covered a large range of sizes, from c. 5 cm LL (length of longest leaf) to 87 cm LL. Because of the ease of access collections were made in flood-tolerant, evergreen *Annona glabra* L. trees, which grow abundantly along the shoreline of Barro Colorado island and adjacent peninsulas. All sampled plants were growing under very similar microenvironmental conditions—detailed descriptions can be found in Stuntz et al. [31]. We carefully removed the entire epiphyte from its substrate and brought it to the laboratory. There, we dismantled the plants leaf by leaf and collected all fine organic material which had accumulated in the leaf axils. Large debris (entire leaves, twigs, parts of fruit, etc.) was discarded and large animals (e.g., spiders, beetles, ants) were not registered either. Samples were immediately fixed in 4–5 % formalin. For each plant, we determined the length of the longest leaf, which has been shown repeatedly to be highly correlated with plant dry mass (e.g., [32]). Sampling was done twice, once in the dry season (March 2009, 18 plants) and once in the wet season (November 2010, 36 plants). The detritus was wet in both cases, because it had rained a few days before the sampling in the dry season in March, which had been preceded by several rainless weeks. The volume of the dry organic material was only quantified for the wet season samples.

### Analysis of samples and species identification

The abundance of the meiofauna (e.g., nematodes, rotifers and crustaceans) and macrofauna (different dipteral larvae and coleopteran larvae) was determined under a stereomicroscope (Zeiss Stemi SV11 Apo, Jena, Germany) at 40× magnification without sieving. The organic material containing minute invertebrates (meiofauna) was preserved in a 4 % formaldehyde solution and stained with 1 % Rose Bengal. At least 50 nematodes were mounted on slides following Seinhorst [33] and subsequently identified whenever possible to species level under a Leitz Dialux microscope (1250×) with differential interference contrast. We used standard identification keys (e.g., [34–36]) and the listed references for species and genera in these books.

Nematode species were assigned to feeding-types (deposit-feeders, epistrate-feeders, suction-feeders and chewers) based on the morphology of their buccal cavity and pharyngeal structure [37]. Deposit-feeding nematodes show an unarmed buccal cavity, only enabling them to ingest particles in the bacterial size-range. Epistrate-feeders possess a small tooth mainly feeding on algae. In contrast, larger suction-feeding or chewing nematodes possess a stylet or large sclerotized teeth, enabling them to prey on a wider range of food items, including invertebrates that are larger than themselves.

### Statistical analysis

Most data analysis was carried out with the program R 2.15.0 [38]. Before the performance of parametric statistics we controlled for homoscedasticity and normal distribution. In order to allow log transformation in the case of zero values, 1 was added to all values, e.g., for some abundance data.

We calculated the Shannon index (H′) for each sample with EstimateS 8.20 [39]. This index is defined as

1 $${\text{H}}^{\prime } = \sum {p_{i} \ln \, \left( {p_{i} } \right)}$$

where *p*_*i*_ is the proportion of the total sample belonging to the *i*th species.

### Field work permission

Permission to work in the Barro Colorado Nature Monument was granted by the Smithsonian Tropical Research Institute. Permits to export the collected animals were granted by the Panamanian authorities (SEX/AP-01-09 and SEX/P-4-11).

## Results

### Plant size and amount of detritus

**Fig. 1:**
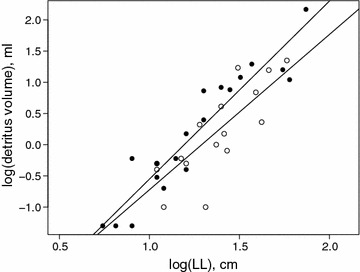
Relationship of detritus volume in ml (*y*) and plant size (expressed as length of longest leaf = LL) in cm (*x*). Note the logarithmic scales. Broad-leaved *Werauhia sanguinolenta* (*filled symbols*) are distinguished from narrow-leaved species (*open symbols*). The regression equations are for log(y) = −3.4 + 2.9 log(x), R^2^ = 0.87, p < 0.001 (*W. sanguinolenta*) and log(y) = −3.2 + 2.5 log(x), R^2^ = 0.62, p < 0.001 (other species)

The volume of detritus, which was only determined in the wet season, scaled with plant size (Fig. 1) in all four species, but the total amount was consistently larger in *Werauhia sanguinolenta* (ANCOVA, p = 0.04, Additional file 1: Table S2). This species has fewer, but much broader leaves than the three other species. Whereas the amount of detritus in smaller plants was quite negligible, it amounted to about 150 ml in the largest individual included in our study.

### Composition of the meiofauna

**Fig. 2:**
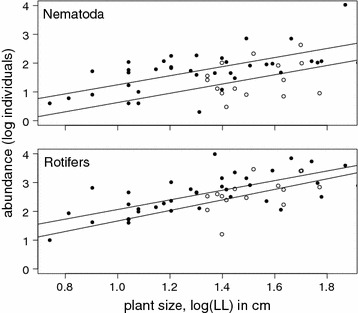
Abundance of nematodes (*upper panel*) and rotifers (*lower panel*) as a function of plant size (expressed as length of longest leaf = LL) in the rainy (*closed symbols*) and dry season (*open symbols*). In both cases, the slopes of the regression lines are significantly smaller than the slope of relationship of plant size and detritus (Fig. 1). Note the logarithmic scale

In terms of individuals, rotifers were by far the most abundant group in both the dry and the wet season, followed by nematodes, annelids and harpacticoid copepods (Fig. 2; Additional file 2: Table S1). In terms of ubiquity, only rotifers, nematodes and mites were found in all samples irrespective of season, although mites were far less abundant than the first two groups. With a single exception (Acari), abundances scaled significantly with the size of the bromeliad tanks for all tested animal groups (using LL as a proxy due to the lack of detritus volume data for the dry season, Table 1). Many groups, e.g., annelids or diptera larvae, were only found in larger plants. This pattern was particularly pronounced for annelids: with a single exception, these were never found in plants ≤20 cm LL, whereas in larger plants they were usually very abundant.

**Table 1:** Results of ANCOVAs on log-transformed data, assessing the effects of plant size and season on the abundance of important animal groups

Group	Plant size	Season
Nematoda	<0.001	0.002
Rotatoria	<0.001	0.057
Harpacticoida	<0.001	0.29
Nauplii	<0.001	0.78
Acari	0.57	0.002
Annelida	<0.001	0.81

Given are the respective p-values. None of the interaction terms was significant (p > 0.05). Individual data for all animal groups and plant specimens are given in Additional file 2: Table S1

Notably, the increase in abundance was significantly smaller than the increase in detritus, i.e., animal densities were substantially lower in larger plants. For example, while the average density of rotifers in small *W. sanguinolenta* plants (<10 cm LL) was 963 ± 630 individuals ml^−1^ detritus (±SD, n = 4), their density in the largest plants (>50 cm LL) was almost one order of magnitude lower (132 ± 180 individuals ml^−1^ detritus, n = 3).

The effect of season was less consistent (Table 1). Abundances were only significantly higher in nematodes and mites during the wet season. Rotifers tended to show higher abundances (p = 0.057), but in the other groups abundances were indistinguishable among seasons.

### Nematode community composition

We found a total of 89 nematode morphospecies in this study (Table 2). Of these, we identified >1/3 to species, and all but 11 morphospecies at least to genus. Individual plants hosted up to 25 species. In the wet season we found 67 species, of which six species had a relatively high abundance of >5 % of the total: *Prismatolaimus* cf *intermedius* (14.8 %), *Tylencholaimellus minimus* (14.2 %), *Tripylella* sp (9.4 %), *Aphelenchoides* sp (9.3 %), *Mylonchulus lacustris* (5.4 %) and *Westindicus* sp (5.1 %). Twenty-nine species were very rare (relative abundance <0.2 %). In the dry season we found 43 species, and eight species had a relative abundance >5 %: *Ditylenchus* cf *acutus* (11.7 %), *A.* sp (9.8 %), Diplogasteridae (Sp 1) (8.6 %), *Prismatolaimus* cf *intermedius* (8.6 %), *Heterocephalobus* cf *elongatus* (7.3 %), *Tylenchus* sp 2 (7.2 %), *Diploscapter coronatus* (5.8 %) and *Geomonhystera tripyloides* (5.2 %). Seventeen species were represented by only one or two individuals. Seasonal differences in the nematode faunas were substantial—only two species (*Prismatolaimus* cf *intermedius* and *A.* sp) were dominant during both seasons and only 20 species were common in both seasons.

**Table 2:** Compilation of the nematode taxa found in the tanks of 54 individuals of four bromeliads species growing in the lowlands of Panama

Species names	Guild	Wet season		Dry season	
		Ind	% tot	Ind	% tot
*Achromadora* sp	Epistrate-feeder	7	0.3		
*Achromadora tenax* (De Man, 1876)	Epistrate-feeder			1	0.2
*Achromadora micoletzkyi* (Stefanski, 1915)	Epistrate-feeder	2	0.1		
*Alaimus* sp 1	Deposit-feeder	2	0.1		
*Alaimus* sp 2	Deposit-feeder	3	0.1		
*Aphelenchoides* sp	Suction-fungi/plant	251	9.3	56	9.8
* Aphelenchoides bicaudatus* (Imamura, 1931)	Suction-fungi/plant	33	1.2		
*Aphelenchus* sp	Suction-fungi/plant	2	0.1		
*Aporcelaimellus* sp	Suction-omnivorous	6	0.2		
*Butlerius* sp	Deposit-feeder	2	0.1		
*Cephalobus* sp 1	Deposit-feeder	7	0.3		
*Cephalobus* sp 2	Deposit-feeder	40	1.5		
*Ceratoplectus* sp	Deposit-feeder	1	0.0		
*Chiloplectus* sp	Deposit-feeder	3	0.1		
*Chromadorina* sp	Epistrate-feeder	3	0.1		
*Diplogaster* sp	Chewer (omnivorous)	1	0.0		
Diplogasteridae (Sp 1)	Chewer (omnivorous)			49	8.6
Diplogasteridae (Sp 2)	Chewer (omnivorous)			2	0.3
Diplogasteridae (Sp 3)	Chewer (omnivorous)			1	0.2
*Diploscapter coronatus* (Cobb, 1893)	Deposit-feeder	4	0.1	33	5.8
*Ditylenchus* cf *acutus* (Khan, 1965)	Suction-fungi/plant	101	3.7	67	11.7
*Dolichorhabditis* sp	Deposit-feeder	1	0.0		
Dorylaimidae (Sp 1)	Suction-omnivorous	3	0.1		
*Ereptonema* sp	Deposit-feeder	1	0.0		
*Ethmolaimus* sp	Epistrate-feeder			1	0.2
*Eudorylaimus* cf *acuticauda* (De Man, 1880)	Suction-omnivorous	24	0.9	2	0.3
*Eudorylaimus* cf *brevis* (Altherr, 1952)	Suction-omnivorous			3	0.5
*Eudorylaimus* sp	Suction-omnivorous	22	0.8	1	0.2
*Eumonhystera simplex* (De Man, 1880)	Deposit-feeder	5	0.2	4	0.7
*Eumonhystera vulgaris* (De Man, 1880)	Deposit-feeder			1	0.2
*Eumonhystera* sp	Deposit-feeder	3	0.1	1	0.2
*Geomonhystera tripyloides* (Andrássy, 1968)	Deposit-feeder			30	5.2
*Geomonhystera villosa* (Bütschli, 1873)	Deposit-feeder	67	2.5		
*Heterocephalobus* cf *elongatus* (De Man, 1880)	Deposit-feeder	80	3.0		
*Heterocephalobus* sp	Deposit-feeder			42	7.3
*Laimaphelenchus* sp	Suction-fungi/plant	2	0.1		
*Malenchus* sp	Suction-fungi/plant	2	0.1		
*Mesodorylaimus* cf *subtiliformis* (Andrássy, 1959)	Suction-omnivorous			13	2.3
*Mesodorylaimus* sp 1	Suction-omnivorous	6	0.2		
*Mesodorylaimus* sp 2	Suction-omnivorous	2	0.1		
*Mesorhabditis* cf *uuglandicola*	Deposit-feeder			4	0.7
*Monhystrella* sp	Deposit-feeder			1	0.2
*Mononchoides* sp	Chewer (omnivorous)			1	0.2
*Mylonchulus brachyuris* (Bütschli, 1873)	Chewer (predator)			7	1.2
*Mylonchulus lacustris* (Cobb in Cobb, 1915)	Chewer (predator)	145	5.4		
*Neoactinolaimus* sp	Suction-omnivorous			2	0.3
*Panagrolaimus* sp	Deposit-feeder	16	0.6	19	3.3
*Paraphelenchus* sp	Suction-fungi/plant	22	0.8		
*Plectus acuminatus* Bastian, 1865	Deposit-feeder	47	1.7	2	0.3
*Plectus* sp 1	Deposit-feeder	76	2.8	1	0.2
*Plectus* sp 2	Deposit-feeder	11	0.4		
*Plectus* cf *minimus* Cobb, 1893	Deposit-feeder	45	1.7	1	0.2
*Plectus longicaudatus* Bütschli, 1873	Deposit-feeder			1	0.2
*Prismatolaimus* cf *intermedius* Bütschli, 1873	Epistrate-feeder	400	14.8	49	8.6
*Prismatolaimus* sp 1	Epistrate-feeder	21	0.8		
*Prismatolaimus* sp 2	Epistrate-feeder	1	0.0		
*Prodesmodora* cf *arctica* (Mulvey, 1969)	Epistrate-feeder			11	1.9
*Prodesmodora loksai* Andrássy, 1989	Epistrate-feeder	91	3.4		
*Rhabdolaimus aquaticus* De Man, 1880	Deposit-feeder	6	0.2		
*Rhabdolaimus terrestris* De Man, 1880	Deposit-feeder	1	0.0		
*Rhabditis* sp	Deposit-feeder	8	0.3		
*Protorhabditis* sp	Deposit-feeder	98	3.6		
Rhabditidae (Sp 1)	Deposit-feeder	21	0.8	15	2.6
Rhabditidae (Sp 2)	Deposit-feeder	2	0.1	25	4.4
Rhabditidae (Sp 3)	Deposit-feeder	1	0.0	18	3.1
Rhabditidae (Sp 4)	Deposit-feeder			8	1.4
*Teratocephalus* sp 1	Deposit-feeder	85	3.1	4	0.7
*Teratocephalus* sp 2	Deposit-feeder	3	0.1		
*Theristus* sp	Deposit-feeder			2	0.3
*Thornia* sp	Suction-omnivorous	19	0.7	3	0.5
*Tripyla* cf *setifera* Bütschli, 1873	Chewer (omnivorous)			3	0.5
*Tripyla* sp	Chewer (omnivorous)			3	0.5
*Tripylella* sp	Chewer (omnivorous)	253	9.4		
*Tripylina arenicola* (De Man, 1880)	Chewer (omnivorous)	18	0.7		
*Tripylina* sp	Chewer (omnivorous)	3	0.1		
*Tylencholaimellus minimus* De Man, 1876	Suction-omnivorous	385	14.2		
*Tylencholaimus* cf *proximus* Thorne, 1939	Suction-omnivorous			17	3.0
*Tylocephalus auriculatus* (Bütschli, 1873)	Deposit-feeder	74	2.7	16	2.8
*Westindicus* sp	Suction-omnivorous	138	5.1		
*Wilsonema* sp	Deposit-feeder	2	0.1		
*Tylenchus* sp 1	Suction-fungi/plant	4	0.1	10	1.7
*Tylenchus* sp 2	Suction-fungi/plant	3	0.1	41	7.2
Tylenchidae	Suction-fungi/plant	1	0.0		
Species 1 (bacteria feeder)	Deposit-feeder	5	0.2		
Species 2 (bacteria feeder)	Deposit-feeder	5	0.2		
Species 3 (bacteria feeder)	Deposit-feeder	1	0.0		
Species 4 (fungi/plant feeder)	Suction-fungi/plant	1	0.0		
Species 5 (bacteria feeder)	Deposit-feeder			1	0.2
Species 7 (bacteria feeder)	Deposit-feeder	5	0.2		

Given are (morpho)-species names, guild classification and abundances in the wet and dry season. Abundance is expressed both as the number of individuals (ind) and as the percentage of a particular species compared to the total number of nematodes per season (% tot). Total abundances were 2704 (wet season) and 572 (dry season) = 572. All species accounting for >5 % of the total individual number are shown in underline

Similar to the trend in individual abundance, species numbers increased with plant size. Also consistent with the abundance data, average species numbers in the wet season were significantly higher than in the dry season (ANCOVA, see Additional file 1: Table S2).

Higher numbers of nematode species (Additional file 3: Figure S1) and increasing individual abundance did not result in a higher diversity in larger plants: the Shannon index varied considerably, but was unrelated to plant size (Additional file 4: Figure S2) or to the amount of detritus (data not shown). Although species diversity tended to be somewhat higher in the wet season, the difference was not significant (*t* test, p > 0.05).

The relative abundances of feeding-types of nematodes differed considerably in the wet and dry season (Table 3). Epistrate-feeder, mainly algae-feeding nematodes, (19.4 vs 10.8 %), omnivorous suction-feeders (22.4 vs 7.2 %) and predators (5.4 vs 1.2 %) all showed higher relative abundances during the rainy season compared to the dry season. In contrast, deposit-feeders (40.0 vs 26.9 %) and suction-feeders on fungi and plants (30.4 vs 15.6 %) were relatively more abundant in the dry season. Only omnivorous nematodes showed almost identical percentages in both seasons (10.2 vs 10.3 %).

**Table 3:** Relative proportions (in %) of feeding-types of nematodes in the tanks of 54 individuals of four bromeliad species growing in the lowlands of Panama, distinguishing collections from the wet and dry season

Feeding-type	Wet season	Dry season
Deposit-feeder	26.9	40.0
Epistrate-feeder	19.4	10.8
Suction-feeder (fungi/plant)	15.6	30.4
Suction-feeder (omnivorous)	22.4	10.3
Chewer (omnivorous)	10.2	10.3
Chewer (predator)	5.4	1.2
Identified nematodes	2704	572

Also given are the total numbers of identified individuals

## Discussion

### Phytotelm size and species richness

An increase in both species and trophic diversity with habitat size is a common observation in ecological systems [40], but the relationship itself does not reveal the underlying mechanism [41]. Assuming that the detritus accumulated in the leaf axils of the bromeliads is a critical resource for the meiofauna, analysing how the amount of detritus changes with plant size may be functionally more relevant than changes in plant size as such. Remarkably, detritus volume scaled with LL cubed (Fig. 1), i.e., the relationship was isometric [42]. We would have anticipated an over-proportional increase of detritus with plant size, because larger plants have a larger catchment area, organic material had more time to accumulate, and water is held for longer periods than in smaller ones allowing more biological activity [26]. The contrasting observations suggest that decomposition is likely to be faster in larger plants, counteracting any increased input rate of organic material. Although both detritus and meiofauna abundance increased with plant size, the relative increase was larger in the former, leading to a drastic decrease in animal density in all studied groups. We cannot offer a satisfying explanation for this observation nor currently predict its functional implications. Repeated sampling is needed to substantiate this finding and experimental manipulation should allow us to detect possible effects on decomposition and other ecosystem processes.

### Species diversity of nematodes

A considerable number of studies has been carried out with a focus on the macrofauna of phytotelms (reviewed e.g., in [9, 20, 43]), which contrast with the meagre information on the meiofauna of these systems. Indeed, this is the first study particularly focusing on the nematode fauna of bromeliad tanks. In total, we identified 89 species of nematodes with diverse assemblages of up to 25 species in a single plant. A high alpha diversity of nematodes between 50 and 100 species is typical for soft and hard substrates of many lakes and streams (e.g., [44–47]). One of the few studies which included a tropical site, with an ecometagenetic approach using 454 pyrosequencing [48], documented a high nematode diversity (and other micro- and mesofauna) within three vertical strata or habitats (soil, litter, and canopy) of rainforests at two contrasting latitudes in the North American meridian (a temperate site in the Olympic National Forest, WA, U.S.A. and a tropical one at La Selva Biological Station, Costa Rica). The authors reported 167 and 214 species, respectively. Boag and Yeates [3] reviewed 134 studies from different ecosystems around the world and identified temperate broadleaf forests with an average of 67 nematode species as the most diverse. Tropical rainforests are seemingly much less species-rich with an average of only 33 species.

### Dominant nematode species

We observed considerable variation in species composition in the dry season (43 species) and the wet season (66 species) in the phytotelms of our tropical lowland site. Only two species were dominant in both seasons, *Prismatolaimus* cf *intermedius* and *A.* sp, while a considerable number of nematode species were present with only very few individuals. In the dry season, a large number of the nematodes were typical for terrestrial or saprobic habitats, e.g., Diplogasteridae, Heterocephalobus and Diploscapter [34, 49, 50]. Many species of these rhabditids like Diplogasteridae and Diploscapter are known to survive not only in saprobic but also in fermented habitats. Clearly, the bromeliad phytotelms offer a habitat for both aquatic and terrestrial nematodes, which at least in part explains why such a large number of species can live in such a relatively small space.

### Feeding-types of nematodes

Nematodes are an excellent group for investigating the distribution of feeding-types because (1) they show very high species numbers and abundances, and (2) they feed on many different food sources, from bacteria to other benthic organisms [51]. During the dry season, the deposit-feeders and the suction-feeders (fungi, plant and omnivorous) were the most abundant feeding-type in bromeliad phytotelms with about 40 %, followed by epistrate-feeders and chewers with about 10 %.

In many lakes and streams the deposit-feeders (mainly bacteria feeders) are dominating (>50 %) [45, 52]. Interestingly, the suction-feeders are below 10 % in most aquatic habitats [44] and values close to 25 % as observed in our data are not very frequently observed [46, 52]. The highest percentage of suction-feeders to date were found in a volcanic lake in Galapagos with 60 % [53]. During the wet season the epistrate-feeders (mainly algae feeders) were twofold higher compared to the dry season and the deposit-feeders have a portion of about 27 %. To conclude, the food web seems to vary strongly with the season.

### Abundance and community composition

Although nematodes and rotifers are the dominant organismal groups of metazoans in many aquatic habitats (e.g., [45, 52, 54]), the reported densities of rotifers and nematodes in the investigated bromeliads are unusually high. This suggests that these organisms play a very important role in the food web of tank bromeliads. Interestingly, very high densities of nematodes were also found in artificial tree-holes in Germany [5]. Brouard et al. [15] studied freshwater organisms (from viruses to macroinvertebrates) in samples taken from 171 tank bromeliads and algae, rotifers and collectors and predatory invertebrates dominated bromeliad food web especially in exposed area. The mean density of rotifers in the six bromeliad species of that study was between 10 and 221 individuals ml^−1^, which is much lower than the densities observed in the current study. Nematodes were not included in the study of Brouard et al. [15]. Jocque and Field [20] investigated 157 bromeliads in Honduras. In total, they found 42 species of meio- and macrobenthos, but nematodes were not mentioned either. We doubt that these were really absent, but were rather not included in the survey.

Overall, our knowledge on the meiofauna of phytotelms is still very sketchy and comprehensive species lists of larger taxonomic groups like ours are very rare. Among the few exceptions there are two studies from Jamaica: Little and Hebert [55] identified nine species of ostracods in 218 bromeliads from 28 sampling sites and Koste et al. [56] identified 17 species of rotifers in terrestrial tank bromeliads. Another study from Mexico [18] documented 61 ciliate species from 39 genera in 52 fresh samples with an average species number of about 7 ciliate species per phytotelm.

## Conclusions

The documented diversity recommends bromeliad tanks as very suitable study systems for questions of community assembly or the relationship of diversity and function. The systems are naturally delimited, highly “replicated”, and easily manipulated. To date, bromeliad tanks are clearly an underutilized resource in this regard. Our study provides a detailed species list, but future studies should study in more detail the underlying mechanisms of community assembly, the temporal dynamics and, last, but not least, the functional implications of diversity for decompositional processes with direct implications for the nutrient supply of the habitat-forming plants.

## Additional files

10.1186/s12898-016-0069-9 Gives the results of an ANCOVA of the data shown in Additional file 3: Figure S1.
10.1186/s12898-016-0069-9 Details the composition of the meio- and macrofauna in 54 epiphytic tank bromeliads in the dry and wet season with information on plant size and amount of detritus (in ml—only in the wet season). For each major animal group the number of individuals is given. The macrofauna is mostly represented by diptera larvae.
10.1186/s12898-016-0069-9 Shows the relationship of nematode species richness and plant size.
10.1186/s12898-016-0069-9 Shows the relationship of nematode diversity (expressed as Shannon diversity index) and plant size.
