# Supplementary material for: What´s in the tank? Nematodes and other major components of the meiofauna of bromeliad phytotelms in lowland Panama
Source: BMC Ecol. 2016 Mar 15;16:9. doi: 10.1186/s12898-016-0069-9 (PMC4791780; doi:10.1186/s12898-016-0069-9)
Supplement: Supplementary file 1 — 10.1186/s12898-016-0069-9 Gives the results of an ANCOVA of the data shown in Additional file 3: Figure S1. [file 12898_2016_69_MOESM1_ESM.docx]

Table S2. Results of an ANCOVA on the effects of plants size (LL) and season on the number of nematode species. Compare Fig. S1.

| Factor | df | F | p |
| --- | --- | --- | --- |
| log(LL) | 1 | 15.5 | <0.001 |
| Season | 1 | 19.6 | <0.001 |
| log(LL) : Season | 1 | 0.58 | 0.45 |
| Error | 49 |  |  |
