# Supplementary material for: What´s in the tank? Nematodes and other major components of the meiofauna of bromeliad phytotelms in lowland Panama
Source: BMC Ecol. 2016 Mar 15;16:9. doi: 10.1186/s12898-016-0069-9 (PMC4791780; doi:10.1186/s12898-016-0069-9)
Supplement: Supplementary file 2 — 10.1186/s12898-016-0069-9 Details the composition of the meio- and macrofauna in 54 epiphytic tank bromeliads in the dry and wet season with information on plant size and amount of detritus (in ml—only in the wet season). For each major animal group the number of individuals is given. The macrofauna is mostly represented by diptera larvae. [file 12898_2016_69_MOESM2_ESM.docx]

Table S1. Composition of the meio- and macrofauna in 54 epiphytic tank bromeliads in the dry and wet season in the tropical lowlands of Barro Colorado Island, Panama. Each plant individual is characterized by species, size (length of the longest leaf in cm, LL) and amount of detritus (in ml – only in the wet season). For each major animal group the number of individuals is given. The macrofauna is mostly represented by diptera larvae.

| Season | Species | LL cm | Detritus ml | Nematoda | Rotatoria | Harpacticoida | Nauplii | Acari | Tardigrada | Planaria | Annelida | Σ Meiofauna | | Macrofauna | |
| --- | --- | --- | --- | --- | --- | --- | --- | --- | --- | --- | --- | --- | --- | --- | --- |
| Dry | *G. monostachia* | 16 |  | 13 | 604 | 381 | 358 | 81 | 0 | 2 | 109 | 1437 | 96 | |  |
| Dry | *G. monostachia* | 22 |  | 12 | 4624 | 14 | 6 | 2 | 2 | 38 | 0 | 4660 | 72 | |  |
| Dry | *W. sanguinolenta* | 22 |  | 26 | 336 | 0 | 0 | 13 | 0 | 0 | 0 | 375 | 0 | |  |
| Dry | *G. monostachia* | 24 |  | 13 | 400 | 340 | 148 | 123 | 0 | 29 | 15 | 1024 | 53 | |  |
| Dry | *W sanguinolenta* | 25 |  | 9 | 16 | 8 | 0 | 11 | 0 | 2 | 0 | 44 | 11 | |  |
| Dry | *W sanguinolenta* | 25 |  | 102 | 336 | 42 | 28 | 7 | 4 | 15 | 8 | 519 | 35 | |  |
| Dry | *T. elongata* | 26 |  | 3 | 248 | 0 | 0 | 2 | 0 | 0 | 0 | 253 | 1 | |  |
| Dry | *G. monostachia* | 28 |  | 36 | 112 | 236 | 32 | 41 | 0 | 0 | 28 | 457 | 2 | |  |
| Dry | *W. sanguinolenta* | 31 |  | 8 | 296 | 29 | 32 | 63 | 1 | 7 | 11 | 429 | 54 | |  |
| Dry | *T. elongata* | 33 |  | 212 | 2912 | 0 | 0 | 9 | 0 | 0 | 0 | 3133 | 7 | |  |
| Dry | *G. monostachia* | 41 |  | 83 | 772 | 10 | 0 | 8 | 0 | 0 | 0 | 873 | 0 | |  |
| Dry | *W. sanguinolenta* | 43 |  | 7 | 568 | 0 | 5 | 3 | 0 | 2 | 14 | 583 | 3 | |  |
| Dry | *W. sanguinolenta* | 43 |  | 26 | 170 | 84 | 119 | 11 | 1 | 14 | 47 | 411 | 32 | |  |
| Dry | *T. elongata* | 50 |  | 428 | 2528 | 0 | 0 | 9 | 0 | 0 | 0 | 2965 | 0 | |  |
| Dry | *W. sanguinolenta* | 50.4 |  | 99 | 2596 | 82 | 12 | 3 | 0 | 0 | 322 | 2792 | 21 | |  |
| Dry | *W. sanguinolenta* | 59 |  | 9 | 692 | 47 | 40 | 11 | 0 | 0 | 8 | 799 | 77 | |  |
| Dry | *W. sanguinolenta* | 83 |  | 103 | 784 | 57 | 39 | 45 | 0 | 0 | 933 | 1028 | 46 | |  |
| Dry | *W. sanguinolenta* | 87 |  | 377 | 3360 | 19 | 16 | 12 | 0 | 0 | 301 | 3784 | 27 | |  |
| Wet | *W. sanguinolenta* | 5.5 | 0.05 | 4 | 10 | 0 | 0 | 6 | 0 | 0 | 0 | 20 | 0 | |  |
| Wet | *W. sanguinolenta* | 6.5 | 0.05 | 6 | 86 | 0 | 0 | 12 | 0 | 0 | 0 | 104 | 0 | |  |
| Wet | *W. sanguinolenta* | 8 | 0.6 | 52 | 656 | 1 | 0 | 36 | 0 | 0 | 0 | 745 | 3 | |  |
| Wet | *W. sanguinolenta* | 8 | 0.05 | 8 | 42 | 2 | 0 | 6 | 0 | 0 | 0 | 58 | 1 | |  |
| Wet | *W. sanguinolenta* | 11 | 0.5 | 58 | 456 | 0 | 0 | 15 | 0 | 0 | 0 | 529 | 1 | |  |
| Wet | *G. monostachia* | 11 | 0.5 | 47 | 40 | 0 | 0 | 37 | 0 | 0 | 0 | 124 | 1 | |  |
| Wet | *T. elongata* | 11 | 0.5 | 4 | 176 | 16 | 4 | 29 | 0 | 0 | 0 | 229 | 8 | |  |
| Wet | *W. sanguinolenta* | 11 | 0.3 | 109 | 52 | 0 | 0 | 23 | 1 | 0 | 0 | 185 | 6 | |  |
| Wet | *G. monostachia* | 11 | 0.4 | 17 | 54 | 0 | 0 | 27 | 0 | 0 | 0 | 98 | 2 | |  |
| Wet | *W. sanguinolenta* | 12 | 0.2 | 4 | 98 | 0 | 0 | 13 | 0 | 4 | 0 | 115 | 1 | |  |
| Wet | *T. elongata* | 12 | 0.1 | 10 | 121 | 0 | 0 | 12 | 3 | 0 | 0 | 146 | 1 | |  |
| Wet | *W. sanguinolenta* | 14 | 0.6 | 61 | 140 | 213 | 9 | 39 | 1 | 0 | 1 | 463 | 2 | |  |
| Wet | *G. monostachia* | 15 | 0.6 | 117 | 188 | 32 | 6 | 28 | 3 | 2 | 0 | 374 | 3 | |  |
| Wet | *G. monostachia* | 16 | 0.5 | 67 | 1032 | 0 | 0 | 38 | 0 | 0 | 0 | 1137 | 6 | |  |
| Wet | *W. sanguinolenta* | 16 | 1.5 | 73 | 232 | 0 | 0 | 11 | 1 | 0 | 0 | 317 | 0 | |  |
| Wet | *W. sanguinolenta* | 16 | 0.4 | 178 | 104 | 34 | 4 | 32 | 1 | 0 | 0 | 353 | 2 | |  |
| Wet | *T. fasciculata* | 19 | 2.1 | 54 | 590 | 1 | 0 | 11 | 5 | 0 | 0 | 661 | 3 | |  |
| Wet | *W. sanguinolenta* | 20 | 2.5 | 39 | 440 | 0 | 0 | 83 | 1 | 0 | 2 | 563 | 6 | |  |
| Wet | *W. sanguinolenta* | 20 | 7.3 | 185 | 460 | 2 | 0 | 137 | 0 | 0 | 19 | 784 | 55 | |  |
| Wet | *T. fasciculata* | 20.5 | 0.1 | 2 | 128 | 0 | 0 | 11 | 1 | 0 | 0 | 142 | 0 | |  |
| Wet | *T. elongata* | 23.5 | 1 | 45 | 9712 | 78 | 2 | 113 | 0 | 0 | 0 | 9950 | 6 | |  |
| Wet | *G. monostachia* | 25 | 4.1 | 149 | 724 | 0 | 0 | 116 | 1 | 30 | 0 | 990 | 28 | |  |
| Wet | *W. sanguinolenta* | 25 | 8.3 | 12 | 1426 | 337 | 114 | 24 | 0 | 0 | 17 | 1913 | 69 | |  |
| Wet | *T. fasciculata* | 26 | 1.5 | 109 | 572 | 36 | 5 | 146 | 6 | 0 | 0 | 874 | 1 | |  |
| Wet | *T. elongata* | 27 | 0.8 | 45 | 319 | 0 | 0 | 53 | 0 | 0 | 0 | 417 | 24 | |  |
| Wet | *W. sanguinolenta* | 28 | 7.6 | 30 | 2250 | 156 | 13 | 99 | 0 | 0 | 7 | 2548 | 32 | |  |
| Wet | *G. monostachia* | 31 | 17.1 | 714 | 1419 | 13 | 12 | 40 | 0 | 19 | 7 | 2198 | 224 | |  |
| Wet | *W. sanguinolenta* | 32 | 12 | 82 | 812 | 1567 | 320 | 91 | 3 | 0 | 3 | 2875 | 64 | |  |
| Wet | *W. sanguinolenta* | 37 | 19.6 | 90 | 227 | 895 | 96 | 101 | 1 | 0 | 343 | 1410 | 165 | |  |
| Wet | *T. fasciculata* | 39 | 6.9 | 95 | 2608 | 16 | 5 | 2 | 0 | 0 | 18 | 2726 | 18 | |  |
| Wet | *T. elongata* | 42 | 2.3 | 47 | 114 | 109 | 25 | 10 | 1 | 0 | 1 | 306 | 29 | |  |
| Wet | *T. fasciculata* | 46 | 15.7 | 708 | 6988 | 22 | 4 | 33 | 0 | 19 | 13 | 7755 | 153 | |  |
| Wet | *W. sanguinolenta* | 55 | 15.9 | 116 | 5412 | 232 | 62 | 267 | 4 | 0 | 32 | 6093 | 178 | |  |
| Wet | *T. elongata* | 58 | 22.4 | 104 | 958 | 482 | 72 | 26 | 33 | 0 | 1219 | 1675 | 85 | |  |
| Wet | *W. sanguinolenta* | 60 | 11 | 116 | 320 | 31 | 0 | 91 | 2 | 15 | 8 | 560 | 81 | |  |
| Wet | *W. sanguinolenta* | 74 |  | 10570 | 3911 | 107 | 36 | 47 | 0 | 0 | 792 | 14671 | 1136 | |  |
