# Supplementary figures and images for: What´s in the tank? Nematodes and other major components of the meiofauna of bromeliad phytotelms in lowland Panama
Source: BMC Ecol. 2016 Mar 15;16:9. doi: 10.1186/s12898-016-0069-9 (PMC4791780; doi:10.1186/s12898-016-0069-9)

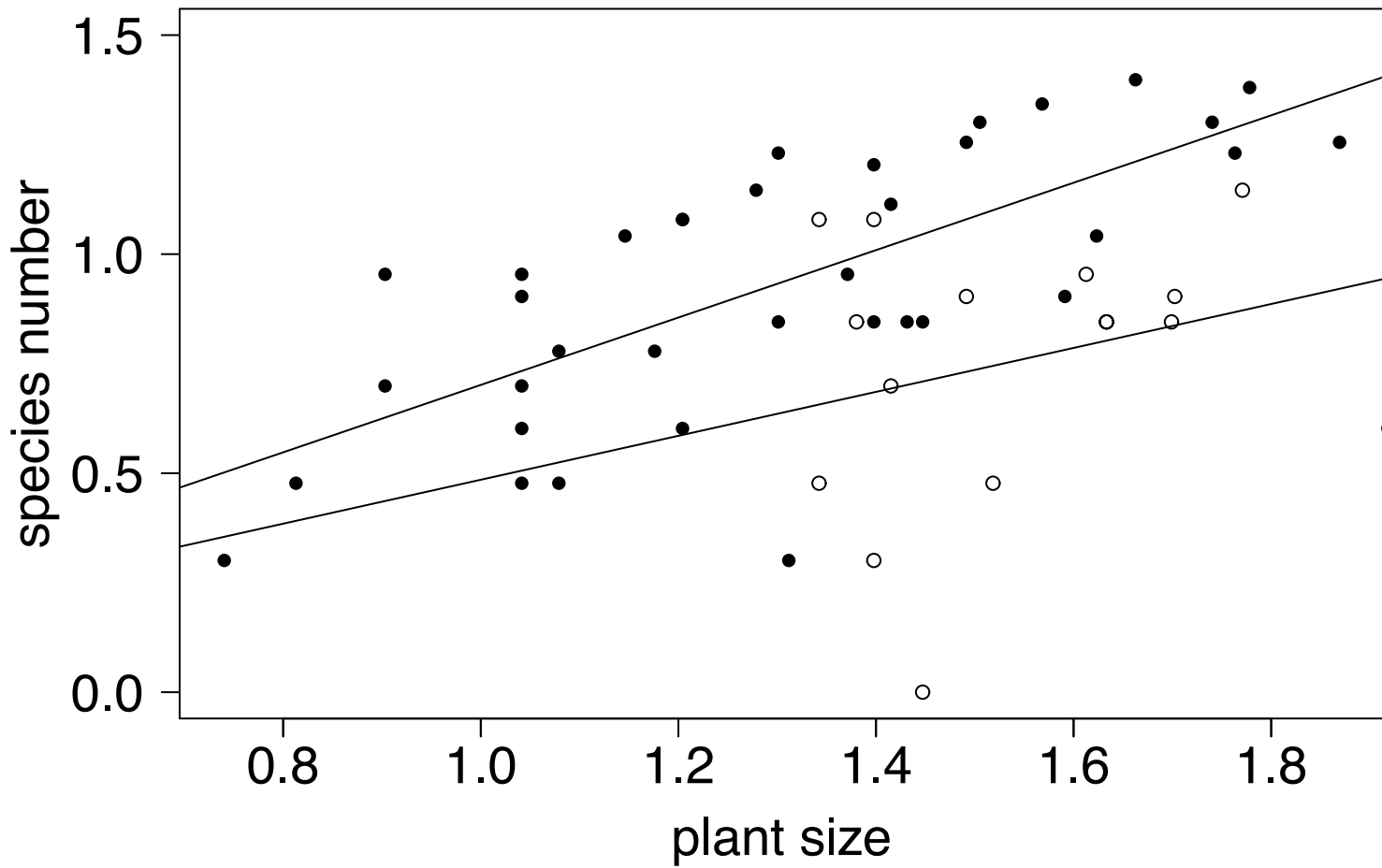

Supplement: Supplementary file 3 — 10.1186/s12898-016-0069-9 Shows the relationship of nematode species richness and plant size. [file 12898_2016_69_MOESM3_ESM.pdf]

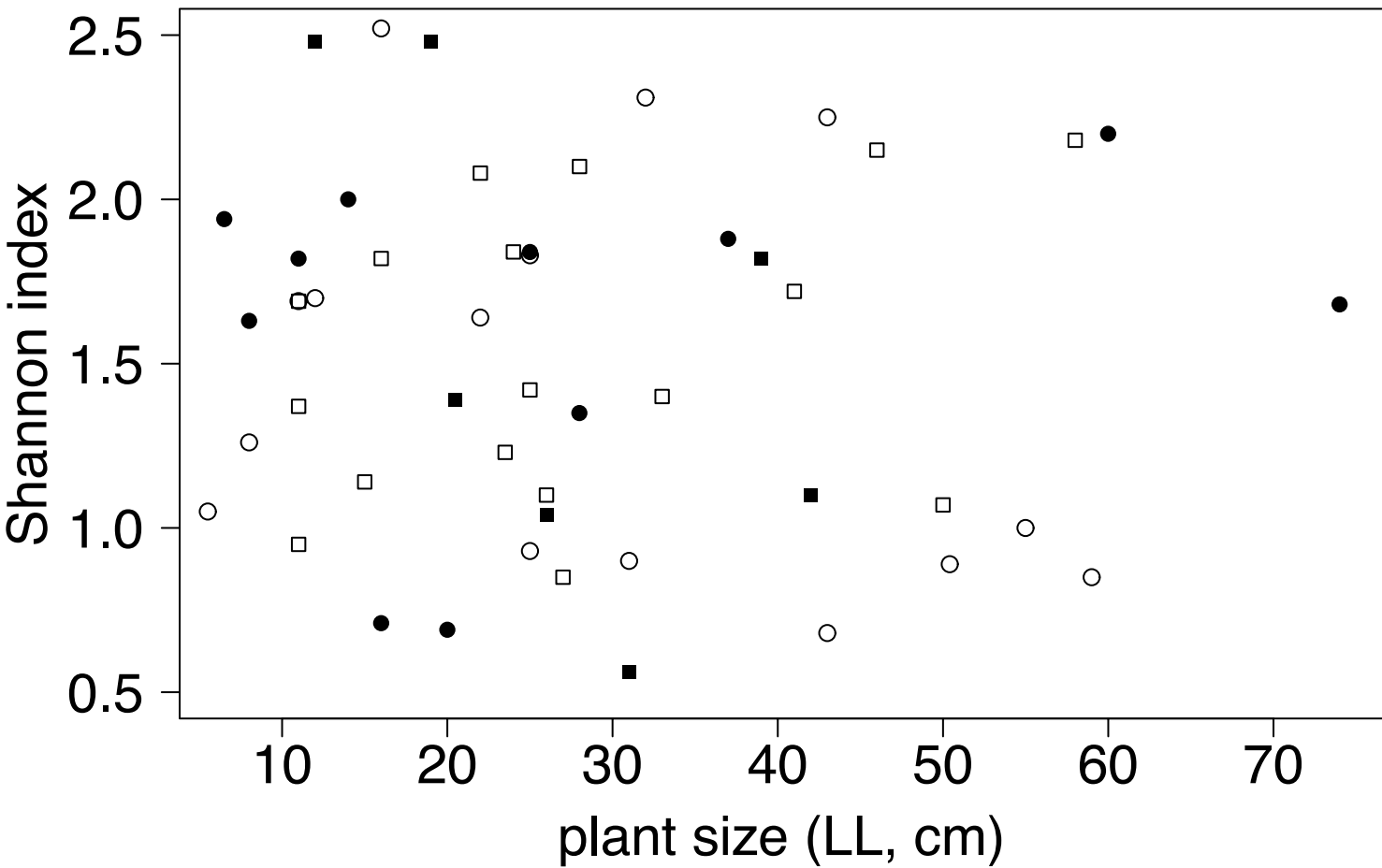

Supplement: Supplementary file 4 — 10.1186/s12898-016-0069-9 Shows the relationship of nematode diversity (expressed as Shannon diversity index) and plant size. [file 12898_2016_69_MOESM4_ESM.pdf]
